# Knowledge, attitude, and practice of diabetes in patients with and without sight-threatening diabetic retinopathy from two secondary eye care centres in India

**DOI:** 10.1186/s12889-023-17371-3

**Published:** 2024-01-02

**Authors:** Shahina Pardhan, Rajiv Raman, Anupama Biswas, Durgasri Jaisankar, Sanjiv Ahluwalia, Raju Sapkota

**Affiliations:** 1 Vision and Eye Research Institute, School of Medicine, Anglia Ruskin University, Young Street, Cambridge, CB1 2 LZ UK; 2 Shri Bhagwan Mahavir Vitreoretinal Services, Sankara Nethralaya, Chennai, Tamil Nadu India; 3 Department of Ophthalmology, Kurseong Sub-Divisional Hospital, Darjeeling, India; 4 School of Medicine, Anglia Ruskin University, Chelmsford, CM11SQ UK

**Keywords:** Diabetic retinopathy, Knowledge, attitude, and practice, Non-sight threatening DR, Questionnaire, Sight-threatening DR

## Abstract

**Background/Aims:**

Good knowledge, Attitude, and Practice (KAP) of diabetes influence its control and complications. We examined the KAP of diabetes in patients with sight-threatening diabetic retinopathy (STDR) and non-sight-threatening diabetic retinopathy (NSTDR) attending two different referral hospitals in India.

**Methods:**

400 consecutive patients (mean age = 58.5 years ± 10.3) with diabetic retinopathy attending retina referral clinics in Chennai (private) and Darjeeling (public) were recruited. A validated questionnaire on diabetic KAP was administered in English or the local language. Data were analysed using an established scalar-scoring method in which a score of 1 was assigned to the correct answer/healthy lifestyle and 0 to an incorrect answer/unhealthy lifestyle/practice. Clinical data included fasting blood sugar, blood pressure, retinopathy, and visual acuity. Retinopathy was graded as STDR/NSTDR from retinal images using Early Treatment of Diabetic Retinopathy Study criteria.

**Results:**

Usable data from 383 participants (95.8%) were analysed. Of these, 83 (21.7%) had STDR, and 300 (78.3%) had NSTDR. The NSTDR group reported a significantly lower total KAP score (mean rank = 183.4) compared to the STDR group (mean rank = 233.1), z = -3.0, *p* < 0.001. A significantly greater percentage in the NSTDR group reported to being unaware that diabetes could affect eyes, did not know about possible treatment for DR, and checked their blood sugar less frequently than once a month.

**Conclusion:**

Patients who had not developed STDR had poorer KAP about diabetes and diabetes-related eye diseases. This is an important issue to address as the risk of their progressing to STDR is high unless appropriate steps to improve their knowledge/awareness and lifestyle practice are introduced early.

## Introduction

Diabetes is one of the fastest-growing public health diseases. In 2021, it was estimated that there were more than 463 million people living with diabetes worldwide, and this number is projected to rise to 643 million by 2030 [1]. It is also known that South Asians are at an increased risk of diabetes and its complications [2, 3], for which the lack of knowledge/awareness about the disease [4, 5], poor self-care practice [5–7], cultural myths [8, 9], rapid urbanization [3], and increased visceral fat [2, 8] have been identified as contributing risk factors. According to the International Diabetes Federation, more than 74 million adults were living with diabetes in India in 2021, i.e., one in every 12 adults [1], and this number is projected to rise to more than 123 million by the year 2040 [10], making India the epicentre of diabetes prevalence.

Diabetic retinopathy (DR) is a significant microvascular complication of diabetes [11]. It is the leading cause of blindness among the working-age population worldwide [12]. Factors such as age, duration of diabetes, South Asian or Black ethnicity, poor control of diabetes, and high blood pressure and cholesterol levels are strongly associated with the high risk and the rate of progression of DR [10, 13].

The prevalence of DR is growing rapidly in India. A recent study in individuals with diabetes over the age of 40 years estimated the national prevalence of any form of DR to be 12·5%, and sight-threatening DR to be 4% [14]. In individuals aged 50 years or above, a higher prevalence of DR (17%) was reported with a narrow difference between rural (14%) and urban (17.4%) settings [15, 16]. Studies conducted in patients attending tertiary care hospitals suggest a range of DR that varied between 32%-63% [17, 18].

Knowledge, Attitude, and Practice (KAP) of diabetes are known to influence the control of DR [19]. Poor KAP encompasses factors including (but not limited to) not knowing whether diabetes can affect the eyes, being unaware of the potential benefits of attending diabetes and DR screening services, forgetting to take medication, and not attending screening services for reasons that range from reduced access to healthcare facilities, financial problems, and underlying co-morbid health conditions [20]. Lifestyle practices including engaging in physical exercise, eating a healthy diet, and refraining from or minimising smoking and alcohol consumption, play an important role in controlling blood sugar, blood pressure, and cholesterol levels. Additionally, self-care practices such as adherence to prescribed medication regimen, and regular monitoring of blood glucose, cholesterol, and blood pressure levels regularly are also vital for ensuring good control of diabetes and DR [10].

Low health literacy around diabetes and DR has been widely reported in both the general and the diabetic population in Indian and other South Asian communities [17, 21–24]. In a sample of 288 Indian people with diabetes, only around 4.5% were found to have good knowledge about how to control DR, and 61% did not have periodic eye examinations, of which 38.5% were not even aware of the benefits of having regular retinal screening [18]. Rani et al. [21] reported that 63% of the rural Indian population did not know that DR is a complication of uncontrolled diabetes. In addition to low health literacy, lower uptake of retinal examination with dilated pupils is reported to be the most important risk factor for sight-threatening diabetic retinopathy in India [25].

Although KAP pertaining to diabetes and DR has been studied extensively in the Indian population, there are no data to date that have compared overall KAP and individual KAP components in patients with sight-threatening diabetic retinopathy (STDR) and non-sight-threatening diabetic retinopathy (NSTDR), which the present observational study aims to examine. The findings will be important in exploring whether there is a need for interventions and close monitoring of patients with NSTDR well before they develop STDR. If differences between the two groups (STDR and NSTDR) exist, then specific groups would need to be targeted in order to reduce the high-risk burden of STDR and blindness in India.

## Methods

### Study population

Four hundred consecutive patients who were referred to the diabetic retinopathy clinic from general eye OPD at Sankara Nethralaya Eye Hospital (*n* = 200), Chennai (located in the South-Eastern region of India), and the other at Kurseong sub-divisional hospital (*n* = 200) in Darjeeling (located in the North-Eastern region of India) were enrolled. A previous study from India estimated the prevalence of DR to be approximately 13% [14]. With a 15% prevalence rate, precision error of 5% and type 1(α) error of 5%, the required sample size would be 174. We examined a slightly larger sample size of 200 patients from each recruitment centre in this study. The sample size used in each centre is also comparable to previous single-centre studies carried out among Indian and Nepali diabetic patients [5, 26] that examined KAP of diabetes control. The study protocol was approved by the Institutional Review Boards of Sankara Nethralaya Eye Hospital and Kurseong sub divisional Eye Hospital. All participants provided informed consent for taking part prior to their inclusion in the study. All methods were carried out in accordance with relevant guidelines and regulations that adhered to the tenets of the declaration of Helsinki. Information that could identify individual participants during or after data collection was not recorded. Participants with either Type 1 or Type 2 diabetes and with any stage of diabetic retinopathy (DR) were included. Participants with ocular pathologies such as advanced glaucoma, matured cataract, and severe uveitis in whom retinopathy grading would be difficult were excluded. Data were collected between March 2016 and February 2017.

### Procedures

A validated questionnaire adapted from our previous studies [5, 13, 26] was administered either in English or the native languages such as Hindi, Tamil or Nepali (people in Darjeeling often prefer to also speak in the Nepali language) as preferred by the participant. The questionnaire contained information about demographics, KAP of diabetes, treatment history of diabetes and DR, in addition to patient’s perception about whether diabetes restricted their everyday activities and if their diabetes was well controlled. The answers provided in native language were translated back into English by an independent investigator who was fluent in both languages. There were three questions on knowledge (K), two questions on attitude (A), and five questions on practice (P), each with multiple choice answers (e.g., ‘yes’, ‘no’, ‘I do not know’). The answers were re-labeled into binary form and analyzed using the scalar-scoring method described elsewhere [27]; scores of 1 for a correct answer/healthy lifestyle practice and 0 for an incorrect answer/unhealthy lifestyle practice were assigned. The maximum possible KAP total score was 10 per questionnaire, and the minimum was 0. The questionnaire was piloted on a small sample of patients with DR (*n* = 15) who were not included in this study.

All participants also underwent a comprehensive eye examination including visual acuity, slit lamp examination, dilated fundus examination, and retinal photography. The slit lamp examination allowed to evaluate ocular conditions such as matured cataract and uveitis that can lead to hazy ocular media preventing gradable retinal photographs to be captured. Retinal photographs were graded by two highly experienced retina specialists following the ETDRS criteria. Intergrader agreement between the two retina specialists was 90%, which was ascertained through a sample of 50 retinal photographs. Fasting blood sugar (FBS) was recorded from all the participants on the day of data collection. Information about systemic health and previous ocular history was obtained from medical records when the visits to the eye clinic took place.

DR severity was categorized according to ETDRS grading criteria. Retinopathy severity of moderate non-proliferative diabetic retinopathy or worse, and/or the presence of diabetic macular edema characterised by retinal thickening within 2-disc diameters of the centre of the macula [28] was categorized as STDR, otherwise assigned as NSTDR.

### Statistical analysis

Statistical analyses were carried out using the IBM SPSS Statistics 28.0.1 version 3 software. Any associations between KAP score and the presence or absence of STDR were examined using the chi-square (χ^2^) (Fischer’s Exact) test. Quantitative data such as FBS, visual acuity, and blood pressure were compared between the participant groups using independent samples t-test. A Mann–Whitney U test was performed to compare the KAP scores between the participant groups and the recruitment centres. Cross-sectional reporting guidelines of ‘Strengthening the Reporting of Observational Studies in Epidemiology (STROBE) were followed [29].

## Results

### Demographics

In total, 295 males (mean age = 60.0 ± 12.4 years) and 105 females (mean age = 59.5 ± 12.0 years) took part, but for 17 participants (4.2%), it was not possible to grade the retinopathy level due to hazy ocular media that precluded grading of DR severity and were thus excluded from the analysis. Demographic data included age, gender, education level, and duration, treatment, and type of diabetes (Table 1). Of the total participants (*n* = 383) included in the analysis, 83 (21.7%) had STDR (64 males- 77.1%, 19- 22.9% females) with a mean age of 59.5 ± 11.4 years and 300 (78.3%) had NSTDR (217 males- 72.3%, 83–27.7% females) with a mean age of 59.6 ± 12 years. Participant group did not differ significantly in mean age (t(381) = 0.06, *p* = 0.95) and was not associated significantly with gender (χ^2^ = 0.7, *p* = 0.40). Data on the education level showed that 3.3% of the participants in the STDR and 2.4% in the NSTDR group were illiterate, which did not associate significantly with the participant groups (χ^2^ = 2.2, *p* = 0.34). A significantly greater proportion of participants in the STDR group (72.3%) had a diabetic duration of more than 5 years compared to 38.7% in the NSTDR group (*p* < 0.001).

**Table 1:** Summary of demographics of study participants between groups

Variable	NSTDR		STDR		*P* value
	(*N* = 300)		(*N* = 83)		
	Mean	SD	Mean	SD	**Independent t-test**
**Age** (years)	59.6	12.0	59.5	11.4	0.9
**Gender**	**Number**	**Percentage**	**Number**	**Percentage**	**Chi-square test**
Male	217	72.3%	64	77.1%	0.4
Female	83	27.7%	19	22.9%	
**Education level**					0.3
None	10	3.3%	2	2.4%	
Primary/Secondary	213	71.0%	53	63.9%	
University/College	77	25.7%	28	33.7%	
**Family history of Diabetes**					0.4
Yes	199	66.3%	52	62.7%	
No	101	33.7%	31	37.3%	
**Duration of Diabetes**					< 0.001
Up to 5 years	184	61.3%	23	27.7%	
> 5 years	116	38.7%	60	72.3%	
**History of diabetic treatment**					< 0.001
Diet only and or tablets	258	86.0%	39	47.0%	
Insulin/mixed	42	14.0%	44	53.0%	
**History of DR treatment**					< 0.001
Yes	12	4.0%	52	62.7%	
No	288	96.0%	31	37.3%	
**Type of diabetes**					< 0.001
Type 1	12	4.0%	16	19.3%	
Type 2	288	96.0%	67	80.7%	

Out of the total 383 participants, 7.3% had Type 1 diabetes and 92.7% had Type 2 diabetes. STDR was significantly more common in those Type 1 diabetes (57%) and NSTDR was more common in those with Type 2 diabetes (81%) (*p* < 0.001). 22.5% of the total participants self-reported receiving insulin (or combined with tablet/diet control) treatment previously, which was significantly associated with STDR (STDR, 53% vs. NSTDR, 14%) (χ^2^ = 56.8, *p* < 0.001. Around 63% of the participants in the STDR group self-reported having treatment for DR previously compared to 4% in the NSTDR group (χ^2^ = 160.7, *p* < 0.001). It is important to note that not everyone with STDR had previous treatment for STDR, probably as they were attending for the first time, or that their vision had been relatively good until recently.

### Clinical findings

Clinical data such as FBS, blood pressure, and visual acuity are provided in Table 2. The mean FBS levels for patients in the STDR and the NSTDR were 165.9 mg/dL (± 59.6) and 146.6 mg/dL (± 55.7), respectively (t(381) = 2.8, *p* = 0.002). As the mean FBS levels in both groups were above the threshold criteria for a confirmed diagnosis of diabetes (≥ 126 mg/dL), data suggest that both groups of participants presented with poorly controlled diabetes, but it was much more apparent in the STDR group (63.9% in the STDR group and 47% in the NSTDR group). Further analysis of FBS data showed that over half of all the participants (50.7%) who self-reported to be on prescribed medication were found to have higher levels of FBS (≥ 126 mg/dL). When asked whether they thought their diabetes is well controlled, over one-fifth of all the participants (21.6%) reported that it was controlled despite the fact that they had FBS of ≥ 126 mg/dL and this differed significantly between the participant groups. Blood pressure, both systolic and diastolic, did not differ significantly between the STDR and the NSTDR groups. As anticipated, participants with STDR had significantly poorer mean log MAR visual acuity in both their better and worse eyes compared to those with NSTDR (Table 2).

**Table 2:** Comparison of clinical data between the participant groups

Variable	NSTDR		STDR		*P* value
	(*N* = 300)		(*N* = 83)		
	Mean	SD	Mean	SD	Independent t-test
Fasting blood sugar (mg/dl)	146.6	55.7	165.9	59.6	0.002
Systolic blood pressure (mmHg)	138.4	19.7	140.7	19.5	0.4
Diastolic blood pressure (mmHg)	79.6	6.6	80.6	7.9	0.3
Visual acuity measurements (logMAR)					
Better eye	**0.1**	0.2	0.3	0.6	< 0.001
Worse eye	**0.3**	0.3	0.7	0.4	< 0.001

Table 3 provides a summary of data pertaining to KAP of diabetes and its control between the participant groups.

**Table 3:** Summary of KAP data between participant groups

**Questions**	**KAP domain**	**NSTDR**		**STDR**		***P* value (independent samples t-test)**
		**Mean**	**SD**	**Mean**	**SD**	
**How many times in the last year did you forgot to take your diabetic medicine?**	Practice	4.6	6.5	4.5	6.8	0.9
**Is exercise important to control diabetes?**		**Number**	**Percentage**	**Number**	**Percentage**	***P* value (Chi-square test)**
Yes	Knowledge	252	84.0%	71	85.5%	0.6
No		10	3.3%	4	4.8%	
Don't know		38	12.7%	8	9.6%	
**Can diabetes affect eye?**						< 0.001
Yes	Knowledge	78	25.7%	52	62.7%	
No		47	15.7%	6	7.2%	
Don't know		175	58.3%	25	30.1%	
**What is the treatment for diabetic eye disease?**						0.006
None/I do not know	Knowledge	134	44.7%	24	28.9%	
Injection/surgery/LASER/combination		166	55.3%	59	71.1%	
**Do you do physical exercise?**						0.2
Yes	Practice	214	71.3%	53	63.9%	
No		86	28.7%	30	36.1%	
**Do you smoke?**						0.5
Yes	Practice	27	9.0%	5	6.0%	
No		273	91.0%	78	94.0%	
**Do you take alcohol?**						0.1
Yes	Practice	42	13.7%	6	7.2%	
No		258	86.3%	77	92.8%	
**How often in the last year did you have episodes of uncontrolled diabetes for which you had to seek medical help?**						
Nil	Practice	158	52.7%	33	39.8%	0.03
At least once		142	47.3%	50	60.2%	
**How often would you check your blood sugar?**						0.03
At least once a month	Attitude	240	80.0%	75	90.4%	
At least once a year		60	20.0%	8	9.6%	
**Would you worry if you forget to inject insulin/take tablets?**						0.2
Yes	Attitude	178	59.3%	58	69.9%	
No		69	23.0%	15	18.1%	
I do not know		53	17.7%	10	12.0%	

A significantly greater proportion of participants in the STDR group reported that they experienced more frequent episodes of uncontrolled blood sugar for which they had to seek medical help. The STDR groups also reported to checking their blood sugar more frequently (at least once a month) compared to the NSTDR group.

A significantly lower proportion of participants in the NSTDR group (25.7%) were aware that diabetes can affect the eyes compared to those in the STDR group (62.7%) or knew the possible treatments for diabetic eye disease.

A Mann–Whitney U test was performed to evaluate whether total KAP scores obtained by using a scalar-scoring method (described above) differed significantly by the participant groups (Table 4). The KAP total score was found to be significantly lower for the NSTDR group (mean rank = 183.4) compared to the STDR group (mean rank = 233.1), z = -3.0, *p* < 0.001. Among the individual KAP components the difference was statistically significant for ‘knowledge’ and ‘attitude’ scores but not for the ‘practice’ scores. This suggests that, although individuals with STDR exhibited better knowledge and attitude about diabetes and DR, they were not translating this knowledge into practice any differently than those with the NSTDR.

**Table 4:** Results of Mann–Whitney test on total and individual KAP scores between the participant groups

Score	Participant group	Total number of participants	Mean Rank	Sum of Ranks	Mann–Whitney test	
					**z-value**	***p*-value**
Knowledge	STDR	83	247.1	20506	-5.6	< 0.001
	NSTDR	300	176.8	53031		
Attitude	STDR	83	212.5	17641	-2.1	0.03
	NSTDR	300	186.3	55895		
Practice	STDR	83	180.7	14996	-1.1	0.27
	NSTDR	300	195.1	58540		
KAP total	STDR	83	233.1	18520	-3.0	< 0.001
	NSTDR	300	183.4	55016		

Next, we examined how the KAP total score differed with gender, age, duration of diabetes, and visual acuity. Lower KAP scores were significantly associated with female gender, age, being on diet only or on tablet treatment, and lower log MAR visual acuity (*p* ≤ 0.05), but not with the duration, type and the family history of diabetes (*p* ≥ 0.16).

### Comparison between the two recruitment centres

Given the significant heterogeneity in patients' demographics, including age, education, occupation, and socioeconomic status has been reported across different parts of India [30], it would be worth comparing data between our recruitment sites (Table 5). The percentage of STDR differed significantly between Kurseong sub-divisional hospital, Darjeeling (16%), and the Sankara Nethralaya Eye Hospital, Chennai (27%).

**Table 5:** Summary of demographics of study participants between two recruitment centres

Variable	Chennai		Darjeeling		*P* value
	(*N* = 200)		(*N* = 183)		
	Mean	SD	Mean	SD	**Independent samples t-test**
**Age** (years)	58.5	10.3	60.7	13.4	0.1
**Gender**	**Number**	**Percentage**	**Number**	**Percentage**	**Chi-square test**
Male	124	62.0%	157	85.8%	< 0.001
Female	76	38.0%	26	14.2%	
**Education level**					< 0.001
None	12	6.0%	0	0.0%	
Primary/Secondary	93	46.5%	173	94.5%	
University/College	95	47.5%	10	5.5%	
**Family history of Diabetes**					0.4
Yes	121	60.5%	102	55.7%	
No	79	39.5%	81	44.3%	
**Duration of Diabetes**					< 0.001
Up to 5 years	57	28.5%	150	82.0%	
> 5 years	143	71.5%	33	18.0%	
**History of diabetic treatment**					< 0.001
Diet only and or tablets	140	70.0%	157	85.8%	
Insulin/mixed	60	30.0%	26	14.2%	
**Any history of DR treatment**					< 0.001
Yes	49	24.5%	15	8.2%	
No	151	75.5%	168	91.8%	
**Type of diabetes**					0.05
Type 1	20	10.0%	8	4.4%	
Type 2	180	90.0%	175	95.6%	

A significantly greater proportion of participants from Chennai had a diabetic duration of more than 5 years and were on insulin/mixed treatment, both of which are associated with an increased risk of STDR [13]. Furthermore, a significantly greater % of participants from Chennai were illiterate, had Type 1 diabetes, and had a history of treatment for DR in the past.

Table 6 provides a summary of data pertaining to KAP of diabetes and its control between the recruitment centres.

**Table 6:** Summary of KAP data between the recruitment centres

**Questions**	**KAP domain**	**Chennai**		**Darjeeling**		***P* value (independent samples t-test)**
		**Mean**	**SD**	**Mean**	**SD**	
**How many times in the last year did you forgot to take your diabetic medicine?**	Practice	4.1	8.8	5.1	2.1	0.13
**Is exercise important to control diabetes?**		**Number**	**%**	**Number**	**%**	***P* value (Chi-square test)**
Yes	Knowledge	140	70.0%	183	100.0%	< 0.001
No		14	7.0%	0	0.0%	
Don't know		46	23.0%	0	0.0%	
**Can diabetes affect eye?**						< 0.001
Yes	Knowledge	103	51.5%	36	19.7%	
No		43	21.5%	2	1.1%	
Don't know		54	27.0%	155	79.2%	
**What is the treatment for diabetic eye disease?**						< 0.001
None/I do not know	Knowledge	136	68.0%	22	12.0%	
Injection/surgery/LASER/combination		64	32.0%	161	88.0%	
**Do you do physical exercise?**						< 0.001
Yes	Practice	94	47.0%	173	94.5%	
No		106	53.0%	10	5.5%	
**Do you smoke?**						0.4
Yes	Practice	14	7.0%	18	9.8%	
No		186	93.0%	165	90.2%	
**Do you take alcohol?**						0.1
Yes	Practice	20	10.0%	28	15.3%	
No		180	90.0%	155	84.7%	
**How often in the last year did you have episodes of uncontrolled diabetes for which you had to seek medical help?**						
Nil	Practice	94	47.0%	97	53.0%	0.03
At least once		106	53.0%	86	47.0%	
**How often would you check your blood sugar?**						< 0.001
At least once a month	Attitude	132	66.0%	183	100.0%	
At least once a year		68	34.0%	0	0.0%	
**Would you worry if you forget to inject insulin/take tablets?**						< 0.001
Yes	Attitude	86	43.0%	150	82.0%	
No		61	30.5%	23	12.6%	
I do not know		53	26.5%	10	5.5%	

A significantly greater proportion of participants from Darjeeling knew that diabetes could affect eyes and were also more aware of the possible treatment for diabetic eye disease compared to the Chennai group. The Darjeeling group were also more aware of the importance of physical activity and took part in it. The Darjeeling group also checked their blood sugar levels more frequently and reported that they would be more worried if they forgot to take their diabetic medicine compared to those in Chennai. This may be partly explained by the higher literacy rate found for participants in Darjeeling. It is possible that differences in the healthcare delivery system and its access and the geographical landscape between the recruitment centres may also have contributed to this, which is beyond the scope of the present study but should form a basis for a future study.

A Mann–Whitney U test was performed to evaluate whether the KAP total score differed by the recruitment centres. The results indicated that the Darjeeling group (mean rank = 257.6) had significantly greater KAP total scores than the Chennai group (mean rank = 132.0), z = -11.3, *p* < 0.001 (Table 7). Within each recruitment centre, the KAP total score was found to be significantly lower for the NSTDR group compared to the STDR group (Darjeeling, mean ranks: STDR = 115.9, NSTDR = 88.9, z = -2.8, *p* = 0.005, and Chennai, mean ranks: STDR = 132.0, NSTDR = 87.5, z = -2.8, *p* < 0.001).

**Table 7:** Results of Mann–Whitney test on total and individual KAP scores between the recruitment centres

Total score	Recruitment centre	Total number of participants	Mean Rank	Sum of Ranks	Mann–Whitney test	
					**z-value**	***p*-value**
Knowledge	Chennai	200	165.2	33035	-5.4	< 0.001
	Darjeeling	183	221.3	40501		
Practice	Chennai	200	156.3	31259	-7.0	< 0.001
	Darjeeling	183	231.2	42278		
Attitude	Chennai	200	142.5	28497	-10.3	< 0.001
	Darjeeling	183	246.1	45039		
KAP total	Chennai	200	132.0	26394	-11.3	< 0.001
	Darjeeling	183	257.6	47143		

## Discussion

This cross-sectional study explored KAP in patients with STDR and NSTDR attending retina referral clinics in two tertiary care hospitals, one located in the north-eastern (Darjeeling) and other located in the south-eastern (Chennai) part of India. The study shows that, overall, the total KAP score pertaining to good diabetic control is poorer in patients with NSTDR compared to STDR. This was also shown when the data for two individual centres were analysed separately. Good diabetic control and regular uptake of retinal screening will reduce the risk of NSTDR progressing to the sight-threatening stage, for which knowledge, awareness, and good practice is vital.

Our findings highlight the importance of improving KAP in patients who have not yet progressed to sight-threatening levels. Poor awareness of diabetic control and complications has been highlighted in other parts of the world including in a study from the USA [31], which reported that 55% of patients who had progressed to diabetic macular edema had not been made aware that they had diabetic retinopathy. In that group, 47% of patients reported that it had been more than one year since they had visited a diabetes educator, dietician, or nutritionist, and 40% reported that they had not received an eye examination with pupil dilation in the last year. Additionally, our data also suggest that effectiveness of interventions to improve KAP may be enhanced by targeting female gender, elderly people, those on diet only or on tablet treatment, and those with lower logMAR visual acuity.

Our participants in the STDR group showed better knowledge and awareness, most likely because a significant majority (63%) had needed treatment. In addition, our study showed poor control of blood sugar by participants: 64% of the participants in the STDR group and 47% in the NSTDR group had a mean FBS of ≥ 126 mg/dL. This is concerning since higher levels of FBS are a significant risk factor for the NSTDR to progress to STDR, and for those with STDR to progress to rapid worsening of their vision.

Visual acuity was significantly worse in participants with STDR compared to NSTDR. Our study also showed that a higher percentage (72%) of participants with STDR had a longer duration of diabetes (> 5 years) compared to 39% in the NSTDR group. This is expected as STDR is associated with a longer duration of diabetes [10]. A greater proportion of participants in the NSTDR group (86%) controlled their diabetes with either diet or tablets compared to the 53% in the STDR group who were on either insulin or a combination of insulin and tablets or diet control) treatment. A previous study from China found that 57% of patients attending an eye clinic at Hangzhou in whom the percentage of STDR was found to be very high (80%) were on insulin (or insulin combined with tablet) treatment. It has been reported [32], that delay in insulin treatment if needed was associated with the worsening of DR. We did not collect data on delays in the use of insulin which could inform a future study.

In our study, a significantly greater proportion of participants in the STDR (38.6%) group reported that diabetes restricted their daily activities compared to 14.3% in the NSTDR group. The STDR group also reported experiencing at least one ‘diabetic episode’ for which they had to seek medical help despite the fact they also reported to checking their blood sugar more often (at least once a month).

On comparing the two centres, STDR was present in 21.7% of all the participants with differences between the recruitment centres (Darjeeling, 16% and Chennai, 27%). Significant geographical variation of DR in India has been reported which is associated with a number of factors including variable health-seeking behaviour, and access to healthcare services [17]. The higher percentage of STDR in Chennai may be due to the fact that Sankara Nethralaya Eye Hospital in Chennai is one of the largest tertiary care referral hospitals not just for the south-eastern region but also for the whole of India and South Asia possibly attracting people with worse retinopathy profiles, whereas Kurseong sub-divisional hospital in Darjeeling is a secondary care referral hospital. In addition, participants in Chennai had a longer duration of diabetes, were on insulin treatment, and had lower total KAP scores, all of which are known to be associated with increased risk of STDR [13, 25]. Notwithstanding these differences, the overall message from both centres is that patients with NSTDR reported lower scores of KAP.

Over 73% of our participants were males. Different factors may have contributed to why a large number of male than female participants attended the retina clinics such as women needing to prioritize family and household responsibilities over their own health, which can deter them from seeking healthcare services [33] and them lacking knowledge and awareness about diabetes [34]. It is also noteworthy that we only included those cases who had some level of DR as the data were collected from DR referral clinics. Future research may explore the KAP for patients with any level of DR and without any level of DR. Our study has some limitations. First, the measurement of Knowledge, Attitudes, and Practices (KAP) relied on a brief 10-point questionnaire. The use of a short questionnaire was chosen to mitigate potential fatigue and boredom among participants, considering that they had already spent a significant amount of time in the eye OPD and retina clinics including blood glucose test on the day of examination prior to questionnaire data collection. Nevertheless, we acknowledge that a more extensive questionnaire could have added depth and richness to our data. Our results may not be representative of the general population as the data were obtained from clinical patients attending referral retina clinics. We hypothesise that the differences in diabetic KAP between STDR and NSTDR may be even greater in the general population. This needs to be investigated. Also, there was a significant heterogeneity in our study population between the two recruitment sites for gender, duration of diabetes and the Type of diabetes (for which it was not possible to control in this observational study). It would have been more appropriate to have glycated hemoglobin (HbA1c) levels measured. However, this is not a common practice in many hospitals in India. Also, our study is a cross-sectional study for which causality cannot be assumed.

To summarise, our study demonstrates that overall KAP pertaining to good diabetic control is poorer in patients with NSTDR compared to STDR. Despite the fact that there are geographical variations, this still holds true. In addition, nearly, two-thirds of the total participants in the STDR group and half of those in the NSTDR group had poorly controlled blood sugar levels (≥ 126 mg/dL) and hence were at higher risk of diabetes-related blindness. In order to redress this, it is vital that all patients including those who had no retinopathy or had mild retinopathy are educated early on good diabetic control and regular uptake of retinal screening to reduce the risk of further damage to their eyes. Culturally and linguistically appropriate interventions aimed at increasing awareness around the importance of diabetic control, promoting healthy lifestyle measures, and improving self-help regimens can help control diabetes and reduce the high-risk burden of STDR.

## Data Availability

The data generated may be obtained upon reasonable request to the corresponding author.
